# The first whole genome and transcriptome of the cinereous vulture reveals adaptation in the gastric and immune defense systems and possible convergent evolution between the Old and New World vultures

**DOI:** 10.1186/s13059-015-0780-4

**Published:** 2015-10-21

**Authors:** Oksung Chung, Seondeok Jin, Yun Sung Cho, Jeongheui Lim, Hyunho Kim, Sungwoong Jho, Hak-Min Kim, JeHoon Jun, HyeJin Lee, Alvin Chon, Junsu Ko, Jeremy Edwards, Jessica A. Weber, Kyudong Han, Stephen J. O’Brien, Andrea Manica, Jong Bhak, Woon Kee Paek

**Affiliations:** Personal Genomics Institute, Genome Research Foundation, Osong, 361-951 Republic of Korea; National Institute of Ecology, Seocheon, 325-813 Republic of Korea; The Genomics Institute, Biomedical Engineering Department, UNIST, Ulsan, Republic of Korea; National Science Museum, Daejeon, 305-705 Republic of Korea; Theragen BiO Institute, TheragenEtex, Suwon, 443-270 Republic of Korea; Department Chemistry and Chemical Biology, Molecular Genetics and Microbiology, and Chemical and Nuclear Engineering, University of New Mexico, Albuquerque, New Mexico USA; Department of Biology, University of New Mexico, Albuquerque, New Mexico USA; Department of Nanobiomedical Science & BK21 PLUS NBM Global Research Center for Regenerative Medicine, Dankook University, Cheonan, 330-714 Republic of Korea; DKU-Theragen institute for NGS analysis (DTiNa), Cheonan, 330-714 Republic of Korea; Theodosius Dobzhansky Center for Genome Bioinformatics, St. Petersburg State University St. Petersburg, Petersburg, 199004 Russia; Oceanographic Center, 8000 N. Ocean Drive, Dania Beach, USA; Nova Southeastern University Ft Lauderdale, Fort Lauderdale, Florida 33004 USA; Department of Zoology, Evolutionary Ecology Group, University of Cambridge, Cambridge, UK; Geromics, Ulsan, 689-798 Republic of Korea

**Keywords:** Cinereous vulture, Old world vulture, New world vulture, Transcriptome, Genome, Next-generation sequencing

## Abstract

**Background:**

The cinereous vulture, *Aegypius monachus*, is the largest bird of prey and plays a key role in the ecosystem by removing carcasses, thus preventing the spread of diseases. Its feeding habits force it to cope with constant exposure to pathogens, making this species an interesting target for discovering functionally selected genetic variants. Furthermore, the presence of two independently evolved vulture groups, Old World and New World vultures, provides a natural experiment in which to investigate convergent evolution due to obligate scavenging.

**Results:**

We sequenced the genome of a cinereous vulture, and mapped it to the bald eagle reference genome, a close relative with a divergence time of 18 million years. By comparing the cinereous vulture to other avian genomes, we find positively selected genetic variations in this species associated with respiration, likely linked to their ability of immune defense responses and gastric acid secretion, consistent with their ability to digest carcasses. Comparisons between the Old World and New World vulture groups suggest convergent gene evolution. We assemble the cinereous vulture blood transcriptome from a second individual, and annotate genes. Finally, we infer the demographic history of the cinereous vulture which shows marked fluctuations in effective population size during the late Pleistocene.

**Conclusions:**

We present the first genome and transcriptome analyses of the cinereous vulture compared to other avian genomes and transcriptomes, revealing genetic signatures of dietary and environmental adaptations accompanied by possible convergent evolution between the Old World and New World vultures.

**Electronic supplementary material:**

The online version of this article (doi:10.1186/s13059-015-0780-4) contains supplementary material, which is available to authorized users.

## Background

Vultures play an important role in the ecosystem by consuming animal carcasses, which helps prevent the spread of disease [1]. The term vulture refers to two independently evolved groups of birds of prey, namely the Old World (16 species) and New World (7 species) vultures. While the two vulture groups are members of different avian families (Accipitridae and Cathartidae), they are phenotypically similar and are both obligate scavengers. The cinereous vulture, also known as the monk vulture, or the Eurasian black vulture, is a member of the family Accipitridae [2], and is a carrion bird [3]. The cinereous vulture (*Aegypius monachus*), the largest bird of prey [4], is distributed throughout Eurasia and is an iconic bird in the Far East. Its population is estimated to number 7,200–10,000 pairs globally, with 5,500–8,000 pairs residing in Asia. Over the past two centuries, its numbers have declined across most of its range leading to this species being classified as ‘near threatened’ on the International Union for Conservation of Nature (IUCN) Red List [5]. Recently, several protection and conservation efforts for this species have been developed at a national [6] and international level [7, 8].

Vultures are suspected to have strong immune defense systems compared to other vertebrates due to their scavenging lifestyle [9], suggesting that they have evolved mechanisms to prevent infection by the microbes found in their diet. Despite the potential interest in the immune system of scavenger birds, little is known about the genetic variations and molecular mechanisms involved in the regulation of immune processes in vultures.

The Avian Phylogenomics Project recently sequenced and analyzed 48 avian species to provide insights into avian evolution [10]. Among the species included were two members of the Accipitridae family, the bald eagle (*Haliaeetus leucocephalus*) and white-tailed eagle (*H. albicilla*). Additionally, the project included the turkey vulture (*Cathartes aura*), which is a member of the Cathartidae (New World vulture). However, no whole genome or transcriptome analyses have been reported for any of the Old World vultures. In order to identify the genetic adaptations and possible convergent evolution linked to obligate scavenging, we provide a whole genome and transcriptome analysis of the cinereous vulture which we compared to other avian genomes; including other birds of prey belonging to the family Accipitridae, as well as New World vultures from the family Cathartidae.

## Results and discussion

### Whole genome sequence

The genomic DNA from a cinereous vulture was sequenced using the Illumina HiSeq2000. We obtained 595 million paired reads (total length, 119 Gb) with a read length of 100 bp and an insert size of 346 bp (Additional file 1: Table S1). We performed a *K*-mer analysis (*K* = 17) using the vulture whole genome sequences (Additional file 2: Fig. S1), and we estimate the genome to be approximately 1.13 Gb (Additional file 1: Table S2), which is consistent with the estimates for other birds (1.0–1.2 Gb) [11–15]. As there is no cinereous vulture reference genome, we aligned the DNA reads to the bald eagle reference, which (as a member of the Accipitridae) is one of the closest species to the cinereous vulture and of higher coverage than the white-tailed eagle (another Accipitridae species) [10]. The mapping rate was relatively high (89.61 %), and the mapped reads covered 91.45 % and 89.84 % of the reference genome and protein coding region at 15× or greater depth, respectively (Additional file 1: Table S1). Compared to the bald eagle reference, there were approximately 34 million single nucleotide variations (SNVs) and 2.0 million small insertions or deletions (indels) in the cinereous vulture. The SNVs were composed of 32,093,779 (95.6 %) homozygous and 1,482,652 (4.4 %) heterozygous SNVs (Additional file 1: Table S3).

### Comparative evolutionary analyses

A set of close but distinct species with clear trait differences can let us predict variants that may contain geno-phenotype association information if their whole genomes are compared [16, 17]. For our comparative evolutionary analyses, we generated a DNA consensus sequence of the cinereous vulture by substituting the vulture SNVs detected from the whole genome sequencing data to the bald eagle reference genome. We then identified orthologous genes between the cinereous vulture consensus sequence and the 3,710 orthologous gene clusters from 17 avian genomes (adelie penguin, american crow, bald eagle, chicken, chimney swift, common cuckoo, crested ibis, downy woodpecker, hoatzin, killdeer, little egret, mallard, ostrich, peregrine falcon, rock pigeon, turkey vulture, and white-tailed eagle) previously reported [18], by matching and adding the cinereous vulture genes to orthologous clusters of the bald eagle genes. A total of 48,081 four-fold degenerate sites of the orthologous gene families were used to calculate the divergence time between the cinereous vulture and the other avian species. The divergence time between the cinereous vulture and bald eagle was estimated to be 18 million years ago (MYA) (Additional file 2: Fig. S2). This split is older than the divergence time between the bald eagle and white-tailed eagle (6–7 million years), and much younger than that of the Old World vultures and New World vultures (about 60 million years) [19]; confirming that the cinereous vulture is more closely related to the eagles.

The identification of positively selected genes (PSGs) is a key genomics tool to understand the evolution of species, especially under distinctive environmental pressures. We identified genes evolving under positive selection by performing tests for deviations in the *d*_*N*_/*d*_*S*_ ratio (non-synonymous substitutions per non-synonymous site to synonymous substitutions per synonymous site, ω) and likelihood ratio tests (LRTs) (*P* ≤0.05) [20, 21]. We successfully discovered a suite of positively selected genes in the Accipitrimorphae clade (Accipitriformes and Cathartiformes orders: bald eagle, cinereous vulture, white-tailed eagle, and turkey vulture). A total of seven and 136 genes were identified as PSGs using, respectively, a branch-site model with a 10 % of false discovery rate (FDR) and a branch model (1 < *d*_*N*_*/d*_*S*_ ≤5 was interpreted as positive selection) (Additional file 1: Tables S4 and S5). To investigate the functional enrichments of these PSGs, we used the DAVID functional annotation tool (Additional file 1: Table S6) [22].

In order to investigate the biologically meaningful PSGs of the Accipitrimorphae, we selected the PSGs associated with the digestive system in the KEGG database (Table 1) [23], since it is known that the Accipitrimorphae have extremely acidic stomachs [24, 25]. Among the PSGs of the Accipitriformes, Somatostatin (*SST*) (ω_average_: 0.04840, ω_Accipitriformes_: 1.4515) was involved in the digestive system pathway. *SST* is a strong inhibitor of gastrin release and plays a role in regulating gastric acid secretion [26]. We suggest that *SST* has been functionally selected in the Accipitrimorphae clade.

**Table 1:** Positively selected genes involved in immune defense and digestive system pathways

KEGG category	KEGG pathways	Accipitrimorphae		Cinereous vulture		Turkey vulture	
		Branch model	Branch-site model	Branch model	Branch-site model	Branch model	Branch-site model
Immune system	Hematopoietic cell lineage			*DNTT*			*GP9*
	Complement and coagulation cascades			*C1QB*			
	Platelet activation					*RAP1B*	*GP9*
	Toll-like receptor signaling pathway	*TAB2*					*LY96*
	NOD-like receptor signaling pathway	*TAB2*					
	Cytosolic DNA-sensing pathway			*POLR1D*			
	Natural killer cell mediated cytotoxicity			*FAS*			
	Fc gamma R-mediated phagocytosis						*ARPC2*
	Leukocyte transendothelial migration					*RAP1B*	
	Chemokine signaling pathway					*RAP1B*	
Digestive system	Salivary secretion						*ADRA1B*
	Gastric acid secretion	*SST*					
	Pancreatic secretion					*RAP1B*	
	Vitamin digestion and absorption			*BTD*		*RBP2*	
	Mineral absorption			*CYBRD1*		*SLC31A1, CYBRD1*	*SLC31A1*

The cinereous vulture belongs to the family of Accipitridae together with the bald eagle and white-tailed eagle, while the turkey vulture (*Cathartes aura*) belongs to the family of Cathartidae, which is an obligate scavenger like the cinereous vulture [27]. To identify the convergent and unique adaptations to a scavenging lifestyle [28], we compared the PSGs of the cinereous and turkey vultures using other birds of prey as a reference (bald eagle, peregrine falcon, and white-tailed eagle). Six and 167 genes were identified as PSGs in the cinereous vulture and 196 and 85 PSGs in the turkey vulture using a branch-site model and a branch model, respectively (Additional file 1: Tables S7-S10). For the PSGs of the cinereous and turkey vultures, we also performed a functional clustering analysis using the DAVID tool (Additional file 1: Tables S11 and S12).

We detected nine PSGs that were shared in both the cinereous vulture (Accipitridae) and turkey vulture (Cathartidae) (Table 2). Among the nine PSGs, several genes were associated with immune functions. The *AHSG* gene is involved in endocytosis, which is a key process that allows cells to internalize particulate matters such as micro-organisms and apoptotic cells. The Alpha2-HS glycoprotein (AHSG) promotes endocytosis and possesses opsonic properties, through which a pathogen is marked for ingestion and eliminated by a phagocyte. In addition, the programmed cell death protein six (*PDCD6*), which encodes a calcium-binding protein required for glucocorticoid-induced cell death, was positively selected in the both vultures. It is known that interaction between PDCD6 and DAPK1 (Death-associated protein kinase 1) can accelerate apoptotic cell death. Viral induction of apoptosis occurs when one or several cells of a living organism are infected with a virus, and many viruses encode proteins that can inhibit apoptosis [29]. We speculate that these positively selected genes may play a role in helping the two vulture species combat pathogens encountered in their diet, complementing the role of gastric secretion discussed previously. Taken together, our genome scale variation analyses have provided interesting new targets for further investigation at the transcriptome, proteome, and even epigenome levels; and will enable future experimental studies to conclusively examine the roles of these genes in the adaptive evolution of these species.

**Table 2:** Positively selected genes by branch and branch-site model that were shared in both the cinereous vulture (Accipitridae) and turkey vulture (Cathartidae)

Gene name	Cinereous vulture				Turkey vulture			
	Branch models		Branch-site models		Branch models		Branch-site models	
	M0: one-ratio	M1: free-ratio	2ΔL	*P* value	M0: one-ratio	M1: free-ratio	2ΔL	*P* value
*ZDBF2*	0.64881	2.2872			0.72895	1.3267		
*MIIP*	0.61631	2.1433			0.62069	1.7923		
*TSPO2*	0.42486	1.704			0.45792	1.2814		
*AHSG*	0.51914	1.5601			0.67974	1.8824	8.166016	4.27E-03
*CRYZL1*	0.14953	1.4475					7.21743	7.22E-03
*C14orf159*	0.31294	1.2295					7.897072	4.95E-03
*PDCD6*	0.04673	1.1766					17.16492	3.43E-05
*C8orf33*	0.59386	1.1212			0.70879	1.9528		
*CYBRD1*	0.37151	1.0177			0.61098	1.7848		

M0 denotes *dn/ds* for all the species in the study; M1 denotes *dn/ds* for the lineage leading to the target species; Likelihood ratio tests (LRT) were used to detect positive selection

**Fig. 1:**
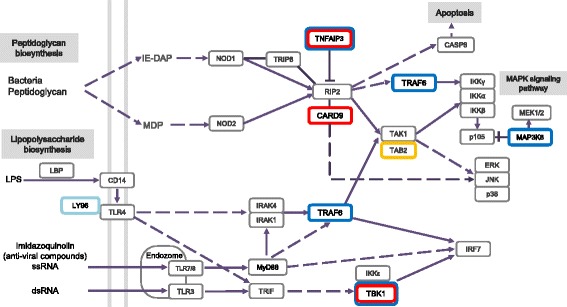
Vulture-specific genetic change in immune system. Genes with vulture-specific amino acid changes (*TNFAIP3*, *CARD9*, *TRAF6*, *MAP3K8*, and *TBK1*) are shown in red (cinereous vulture) and blue (turkey vulture) rectangles. Upregulated gene in the cinereous vulture (*TAB2*) is shown in yellow rectangle. A positively selected gene (*LY96*) in the turkey vulture is shown in a light blue rectangle. The pathway was drawn according to KEGG pathway (NOD-like receptor and Toll-like receptor signaling pathways). The solid lines indicate direct relationships between enzymes and metabolites. The dashed lines indicate that more than one step is involved in a process

**Fig. 2:**
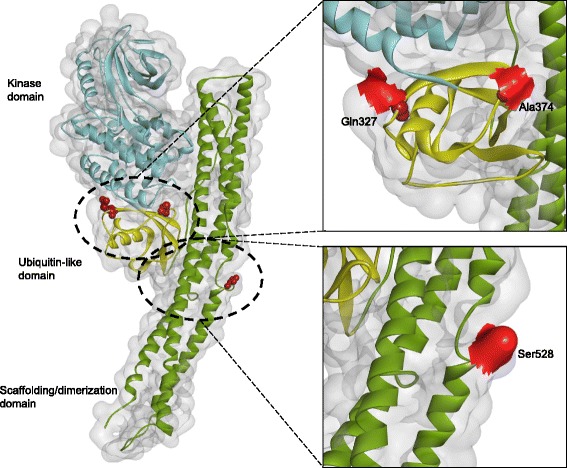
Positions of two vulture-specific amino acid changes in the three-dimensional structure of TBK1. The kinase, ubiquitin-like, and scaffolding/dimerization domains are colored cyan, yellow, and green, respectively. Structural positions of the three amino acids changes in the two vultures are shown in red

If adaptive evolution is episodic and affects only a few crucial amino acids, the application of the PSG criterion is likely to result in the omission of important genes [30]. To overcome this limitation, we investigated the cinereous vulture and turkey vulture-specific amino acid changes in the immune system pathways (Fig. 1) (Additional file 1: Tables S13 and S14). A total of 17 and 24 genes had amino acid changes specific to the cinereous vulture and the turkey vulture, respectively. Among these genes, three were shared only by the cinereous vulture and turkey vulture (Table 3). The Protein Variation Effect Analyzer (PROVEAN) software predicted that the vulture-specific amino acid substitutions observed in all three genes (*PIK3AP1*, *TBK1*, and *TNFAIP3*) are likely to cause alterations to protein function [31]. Of particular interest is TANK-binding kinase-1 (TBK1), which is essential for regulating the response to viral and microbial agents and promotes the development of a cellular antiviral state by activating interferon regulatory factors [32] (Fig. 2). Two of the three vulture-specific amino acid polymorphisms present in TBK1 were predicted as function altering amino acid changes and are located in the ubiquitin-like domain on the exposed surface of the protein. Namely, the positively charged side chain (His) at residue 327 in the cinereous vulture has been replaced by an uncharged side chain (Gln), while the polar side chain (Thr) at residue 364 in the turkey vulture has been substituted with a hydrophobic side chain (Ala). It is known that TBK1 forms several different complexes with a variety of scaffolding molecules, and that the deletion or mutation of the ubiquitin-like domain in TBK1 impairs kinase activation and substrate phosphorylation in cells [33–35]. Therefore, we speculate that the amino acid changes in this gene may contribute to these vultures’ enhanced immune systems. Additionally, the *PIK3AP1* and *TNFAIP3* genes, which also contain functional altering amino acid changes, are involved in B-cell development, antigen presentation, auto-inflammation, and NF-kappa B activation, respectively [36, 37].

**Table 3:** Unique amino acid site changes on sites between two vultures in immune system-related proteins

Protein	Species	Position	Residue	Other species residue	PROVEAN score (Prediction)
PIK3AP1	Cinereous vulture	1	A	T	0.757
	Turkey vulture	68	L	H	−4.913 (Deleterious)
		208	F	Y	−1.206
		232	P	S	−1.225
		398	N	K	−1.674
TBK1	Cinereous vulture	327	Q	H	−5.692 (Deleterious)
		528	S	P	−1.76
	Turkey vulture	374	A	T	−3.225 (Deleterious)
TNFAIP3	Cinereous vulture	446	A	G	−0.857
		742	Q	A,P,L,V	−0.044
	Turkey vulture	243	G	E	−5.456 (Deleterious)
		270	G	E,V	−4.024 (Deleterious)
		590	S	P,L	0.871
		636	T	A	0.385
		643	S	R	0.132
		677	M	T	−2.323

We also investigated the Accipitridae (cinereous vulture, bald eagle, and white-tailed eagle) and Cathartidae (turkey vulture)-specific amino acid changes in the digestive system pathways (Additional file 1: Table S15). A total of eight and 10 genes were identified as unique amino acid changes in the Accipitridae and the Cathartidae, respectively. Among these genes, seven were shared by both the Accipitridae and Cathartidae (Additional file 1: Table S16). Of these, two genes (*KCNJ16* and *SLC26A7*) are involved in the gastric acid secretion pathways (Additional file 2: Fig. S3). KCNJ16 is an inward-rectifier potassium channel which regulates fluid and pH balance, and SLC26A7 is known to play an important role in gastric acidification [38]. Among the five amino acid differences in these genes, two were predicted as function-altering using Polyphen2 [39] and PROVEAN (Additional file 1: Table S17). Additionally, two genes (KCNC1 and KCTD18), which are involved in potassium channel activity, were positively selected in the cinereous vulture, although it was not the case in the turkey vulture. It is known that potassium ions are required for gastric acid secretion [40]. Recently, a vulture metagenome study showed that the facial microbial community was significantly more diverse than that of the hindgut, reflecting the breakdown of dietary DNA in the vulture gastrointestinal tract and pointing to extraordinarily harsh chemical conditions [41]. Taken together, we propose that these vulture-specific amino acid changes in the immune system and gastric acid-associated genes are evidence of the vultures’ enhanced defenses against microbial/viral infections from carcasses and are good candidates for future functional studies.

### Transcriptome analysis

To extend our genomic inferences, we constructed a cDNA library from total RNA isolated from the blood sample of a second cinereous vulture from the same population. Using an Illumina HiSeq2500, a total of 30,118,314 paired reads (approximately 6.0 Gb in length) were produced, with a read length of 100 bp and an insert size of 280 bp. To reduce the effect of sequencing errors, 51,537,752 (85.6 %, approximately 5.15 Gb in length) reads were selected after filtering out ambiguous and low-quality reads. All filtered short-read transcriptome data were assembled using the Trinity *de novo* assembler program [42] with an optimized *K*-mer length of 25. The filtered reads were assembled into 423,702 contigs with a mean length of 3,670 bp (N50 of 6,110 bp), and the contigs were clustered into 352,872 non-redundant unigenes with a mean length of 3,469 bp and an N50 of 6,015 bp (Additional file 1: Table S18).

To validate and annotate the 352,872 assembled unigenes, we searched the GenBank non-redundant (NR) database for homologs of all the assembled unigenes, using the BLASTx program [43] with the criterion E-value ≤ 1.0E-5. A total of 241,347 (68.4 %) out of 352,872 unigenes were homologous to sequences in the NR database. As expected, the BLASTx top-hit species distribution of the vulture unigene was highly enriched in vertebrates, especially among avian species (Additional file 2: Figs. S4 and S5). For a further evaluation of the assembled transcripts, we also examined the expression levels of the assembled vulture unigenes. The number of mapped reads for each annotated unigene was normalized to fragments per kilobase of transcript per million mapped fragments (FPKM). A total of 170,990 unigenes were determined to be expressed (FPKM >0); of those, 73,984 unigenes could be functionally annotated by matching with sequences in the databases. Ultimately, 33,302 functionally annotated and highly expressed unigenes (FPKM ≥1.0) were selected and used in the transcriptome analysis.

A total of 29,774 out of the 33,302 unigenes were functionally clustered into 52 subcategories under three non-mutually exclusive ontologies (cellular components, molecular functions, and biological process) using WEGO [44] (Additional file 2: Fig. S6): 27,632 were assigned to cellular components, 25,572 to molecular functions, and 25,145 to biological processes (Additional file 1: Table S19). Of the cellular components, most unigenes were classified as components of cells (or cell parts), with only a small portion classified as components of virions (or virion parts). Importantly, a considerable number of unigenes were assigned to immune system processes (2,305 unigenes, 7.7 %) and responses to stimulus (5,425 unigenes, 18.2 %) subcategories that could be linked to the vultures’ immune defenses. The numbers are higher than those found in blood lymphocytes transcriptome from the Chinese goose (*Anser cygnoides*) [45], which are 0.3 % and 2.8 % for the immune system processes and the responses to stimulus, respectively. We also analyzed the annotated unigenes using the KEGG automatic annotations server (KAAS) [46]. We obtained KEGG orthology-based annotations for the vulture unigenes from the human, chicken, turkey, and zebra finch. A total of 327 KEGG pathways were identified in the vulture unigenes. The vulture unigenes were most over-represented in metabolic pathways (649 unigenes), biosynthesis of secondary metabolites (162 unigenes), and pathways in cancer (153 unigenes) (Additional file 1: Table S20), which showed a similar pattern found in a crow lung transcriptome [47] (top three over-represented pathways: metabolic pathways, pathways in cancer, and PI3K-Akt signaling pathway).

The Toll-like receptors (TLRs), together with interleukin-1 receptor type 1 (IL-1R) gene family, recognize pathogen components and trigger a signaling cascade through the recruitment of different combinations of toll/interleukin receptor (TIR)-domain containing adaptor proteins [48]. TLRs are essential in pathogen recognition [49], and a previous study reported that *TLR1* in the Griffon vulture (*Gyps fulvus*), an Old World vulture, has a unique gene structure (with respect to the length of the ectodomain, number and position of leucine-rich repeat [LRRs] motifs, and *N*-glycosylation sites) relative to orthologous genes from other organisms [9]. These features have possible functional implications and we confirmed that the *TLR1* unigene of the cinereous vulture has identical LRR motifs and *N*-glycosylation sites (Additional file 2: Fig. S7).

Additionally, we compared the gene expression levels of the cinereous vulture to those of blood lymphocytes from a Chinese goose and blood from four Eurasian siskins (*Spinus spinus*) [50] (Additional file 1: Table S21). We examined the expression levels of immune system associated genes and identified differentially expressed genes that satisfy the log2 fold-change of at least 2.0 and FDR less than or equal to 0.1. A total of four immune system associated genes (*TAB2*, *STAT3*, *CYLD*, and *NFYA*) were significantly highly expressed in the cinereous vulture, compared to the others (Additional file 1: Table S22). The TAB2 protein is a mediator of TAK1 activation in the interleukin-1 signaling transduction pathway. Expression of TAB2 induces JNK (c-Jun N-terminal kinase) and NF-kappaB activation, which upregulate the expression of many proinflammatory genes [51]. The CYLD protein plays an important role in the regulation of pathways leading to NF-kappaB activation [52]. Also, it is known that STAT3 mediates cellular responses to interleukins and growth factors, and STAT3 mediates the expression of a variety of genes in response to cell stimuli, and thus plays a key role in apoptosis.

### Genetic diversity and population history

**Fig. 3:**
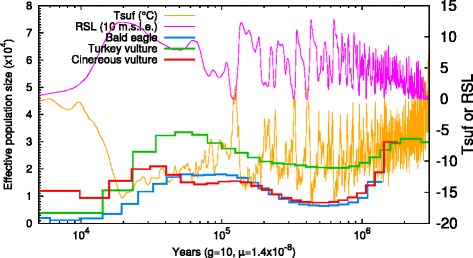
Demographic history of the cinereous vulture. g, generation time (years); μ, mutation rate per site per generation time; Tsuf, atmospheric surface air temperature; RSL, relative sea level; 10 m.s.l.e., 10 m sea level equivalent

The demographic history of a species can be reconstructed from deeply sequenced genomes [53]. We first calculated the level of the genomic diversity of cinereous vulture by measuring the rate of heterozygous SNVs found in the genome. The genomic diversity was found to be 0.00132, which is comparable to the diversity observed in the turkey vulture (0.00148) and much higher than that of humans (0.00069) [54] and bald eagle (0.00053). We also inferred the demographic history of the cinereous vulture using the pairwise sequentially Markovian coalescent (PSMC) model [55] (Fig. 3). Between 1,000,000 and 200,000 years ago, the cinereous vulture and the bald eagle showed similar effective population sizes. However, during the late Pleistocene (approximately 110,000 to 12,000 years ago), the effective population size of the cinereous fluctuated markedly, whereas the bald eagle and the turkey vulture were relatively stable. In particular, the cinereous vulture population experienced a strong bottleneck between 110,000 and 70,000 years ago, whereas the bald eagle and the turkey vulture showed relatively stable and increasing population sizes, respectively.

## Conclusions

Our study provides the first whole genome and *de novo* transcriptome analyses of the cinereous vulture, along with comparative evolutionary analyses with other avian genomes. The comparative analyses revealed likely signatures of selection in cinereous vulture genes associated with the immune response and gastric acid secretion. Together with previous analysis of vulture metagenomes [41], our results suggest that the acidic gastrointestinal tract of vultures is a strong filter of the microbiota ingested from decaying carcasses, which likely plays a role in enabling the vulture’s scavenging lifestyle. A comparison to the turkey vulture revealed a number of possible instances of convergent evolution between Old and New World vultures, in line with the recent observation of convergent evolution (adaptive convergence) based on comparative genomics data [10, 19]. At the same time, it is important to note that convergence might be non-adaptive [56], and simply driven co-evolutionary interactions between sites, with the process at one site changing other coupled sites [57]. Therefore, with additional genomic data, the candidate genes identified in this study can be further investigated to test the possible roles of adaptive versus non-adaptive convergence in this system. While functional studies of candidate genes will be needed to confirm the role of individual genes, this first analysis of the cinereous vulture genome and transcriptome provides insights into the genetic adaptations that have allowed this lineage to successfully occupy such a challenging niche.

## Methods

### Sample preparation and sequencing

Blood samples from two cinereous vultures were acquired from the Korean Association for Bird Protection under the Cultural Heritage Administration (Korea) Permit. DNA was extracted using a QIAamp DNA Mini Kit (Qiagen, CA, USA). The amount of DNA was quantified by fluorometry using a Qubit dsDNA Assay Kit (Invitrogen, CA, USA) and an Infinite F200 Pro NanoQuant (TECAN, Männedorf, Germany). High-molecular weight genomic DNA was sheared to approximately 500 bp using a Covaris S2 Ultrasonicator, and then used in the preparation of whole-genome shotgun libraries according to Illumina’s library preparation protocols (Illumina, CA, USA). The efficacy of each step of library construction was ascertained using a 2100 Bioanalyzer (Agilent Technologies, CA, USA). A final dilution of the library was then sequenced on a HiSeq 2000 (Illumina), using the TruSeq Paired-End Cluster Kit v3 (Illumina) and a TruSeq SBS HS Kit v3 (Illumina) for 200 cycles.

Total RNA was extracted from the blood of an additional vulture using the Trizol LS Reagent (Ambion, TX, USA). RNA quality was assessed by analysis of rRNA band integrity on an Agilent RNA 6000 LabChip kit (Agilent Technologies). Prior to cDNA library construction, 2 μg of total RNA was treated with DNase I, and magnetic beads with Oligo (dT) were used to enrich poly (A) mRNA. Next, the purified mRNAs were disrupted into short fragments, and double-stranded cDNAs were immediately synthesized. The cDNAs were subjected to end-repair and poly (A) addition, and then connected with sequencing adapters using the TruSeq RNA Sample Prep Kit (Illumina). Suitable fragments, automatically purified using a BluePippin 2 % agarose gel cassette (Sage Science, MA, USA), were selected as templates for PCR amplification. The final library sizes and qualities were evaluated electrophoretically using an Agilent High Sensitivity DNA kit (Agilent Technologies); the fragment size range was 350–450 bp. Subsequently, the library was sequenced using a HiSeq 2500 sequencer (Illumina) in rapid run mode. Cluster generation was performed, followed by 2 × 100 cycle sequencing reads separated by a paired-end turnaround. Image analysis was performed using the HiSeq control software version 1.8.4.

### Vulture DNA read alignment to bald eagle reference genome

A DNA read was filtered out when average quality was less than Q20 and/or ambiguous nucleotide ratio was higher than 10 %. The clean reads were aligned to the bald eagle genome sequence using BWA ver. 0.6.2 [58] with default options. The *rmdup* command of SAMtools 0.1.18 [59] was used to remove PCR duplicates from the reads. The raw SNVs and indels were called using the mpileup command of SAMtools ver. 0.1.19 with the -Q 30 option followed by the view command with the -cg option. From the raw SNVs, the consensus genome sequences of the cinereous vulture were generated using vcf2fq with -d 8 and -l 2 options. The final SNVs were defined by comparing the consensus sequence to the reference sequence. The raw indels were filtered using varFilter of vcfutils.pl with -d 8 to produce the final indel dataset. To remove gene sequences possibly derived from paralog genes in the vulture or low sequencing depth regions, we filtered out coding sequences (CDSs) using the following criteria; (1) CDSs that contain ambiguous nucleotides ‘N’; (2) CDSs that contain premature stop codons. Among the 16,526 protein coding genes, 8,546 genes were obtained and used in the further analyses. For the *K*-mer analysis, JELLYFISH [60] was used with *K* = 17. Genome size was estimated from the total number of *K*-mers divided by the peak depth of *K*-mer graph.

### Transcriptome analysis and functional gene annotation

The raw sequencing reads contained low-quality reads and adaptors, which were removed as follows: First, reads with >10 % ambiguous bases (represented by the letter ‘N’) or with Q20 < 20 % were removed. If the average quality of the reads was under Q20, reads were also removed. The bases under Q20 were removed at the both end of a read.

*De novo* transcriptome assembly was performed on the remaining high-quality reads using the short-read assembly software Trinity [42]. These resulting contigs were further processed with the sequence clustering software TGICL [61] to produce unigenes. Coding sequence (CDS) were predicted using TransDecoder [62], and functions of unigenes were predicted by DNA and protein sequence homology searches using BLAST and InterProScan v5 [63]. The NCBI Blast 2.2.28+ and NR database were used to search for protein sequence homology. InterProScan v5, consisting of five protein databases (Pfam, Panther, SMART, SuperFamily, and Gene3D) was used to predict the function of unigenes. The FPKM method [64] was used to calculate unigene expression and gene expression levels were analyzed using the RSEM software [65]. The WEGO software was then used to perform GO functional classification of all unigenes and view the distribution of gene functions. Pathway assignments were performed according to the KEGG pathway database, using KAAS.

To compare the gene expression level of the cinereous vulture, Chinese goose, and Eurasian siskins, the RNA reads from the cinereous vulture, Chinese goose, and Eurasian siskins were aligned to the bald eagle, duck, and American crow reference genome using TopHat2 [66], respectively. The number of reads that were mapped to orthologous gene regions were counted using HTSeq-0.6.1 [67], and then normalized by gene length and transcripts per million (TPM) [68]. The genes having less than 1.0 TPM were filtered out. The TPM values were normalized by Trimmed Mean of M-values (TMM) [69] using edgeR [70]. The significance of differential expression between the vulture and siskins was calculated by the exact negative binomial test, and corrected for multiple testing by the Benjamini-Hochberg false discovery rate.

### Molecular evolutionary analyses

To estimate divergence times, we used four-fold degenerate sites of avian one-to-one orthologous families using RelTime-CC version 2.0 [71] with the phylogenetic tree topology of published previously [19]. The evolutionary rates of the each branch were calculated by the resulting tree. The program used a maximum likelihood statistical method and time reversible model. The date of the node between duck and chicken was constrained to 65.8 MYA [19].

PRANK [72] was used to construct multiple sequence alignment among ortholog genes, and the CODEML program in PAML 4.5 was used to estimate the rates of synonymous (*d*_*S*_) and non-synonymous substitutions (*d*_*N*_) and the *d*_*N*_/*d*_*S*_ ratio (ω) [20]. The one-ratio model, which allows only a single *d*_*N*_/*d*_*S*_ ratio for all branches, was used to estimate the general selective pressure acting among all species. A free-ratios model was used to analyze the *d*_*N*_/*d*_*S*_ ratio along each branch. To further examine potential positive selection, the branch-site test of positive selection was conducted [21]. Statistical significance was assessed using likelihood ratio tests (LRTs) with a conservative 10 % false discovery rate (FDR) criterion [73]. The Accipitrimorphae were used as the foreground branch and the other species as background branch. To identify the convergent evolution of the two vultures, the each vulture as foreground was used and the rest of the bird of prey as the background were used.

Function-altering amino acid changes were predicted using PolyPhen2 [39] and PROVEAN v1.1 [31] using the default cutoff values. A 3D modeling structure of the TBK1 was predicted by SWISS-MODEL [74–76].

### Demographic history analysis

To infer the population history of the cinereous vulture, we used the PSMC program using scaffold length ≥50 kb sequence. We performed 100 rounds of bootstrapping and used 1.4 × 10^−8^ substitutions per site per generation [10]. We used 10 years as a generation time as previously reported [77].

### Availability of supporting data

Whole-genome sequence data and RNA sequence data were deposited in the SRA database at NCBI with BioSample accession numbers SAMN02728242 and SAMN02739871, respectively. The SRA of whole genome sequencing can be accessed via reference numbers SRX518112, and the RNA sequence can be accessed as SRX529363. The data can also be accessed through BioProject accession number PRJNA244786 for the whole-genome sequence and PRJNA245779 for the RNA sequence data. The Transcriptome Shotgun Assembly project has been deposited at DDBJ/EMBL/GenBank under the accession GDQP00000000. The version described in this paper is the first version, GDQP01000000.

## Additional files

Additional file 1: Table S1. Sequencing and analysis statistics of the cinereous vulture’s WGS relative to the bald eagle genome. **Table S2** 17-mer statistics. **Table S3** Summary of SNVs and small indel in the cinereous vulture. **Table S4** PSGs list of the Accipitrimorphae using a branch-site model. **Table S5** PSGs list of the Accipitrimorphae using a branch model. **Table S6** Functional annotation chart of PSGs of the Accipitrimorphae. **Table S7** PSGs list of the cinereous vulture using a branch-site model. **Table S8** PSGs list of the cinereous vulture using a branch model. **Table S9** PSGs list of the turkey vulture using a branch-site model. **Table S10** PSGs list of the turkey vulture using a branch model. **Table S11** Functional annotation chart of PSGs of the cinereous vulture. **Table S12** Functional annotation chart of PSGs of the turkey vulture. **Table S13** Unique amino acid changes of the turkey vulture. **Table S14** Unique amino acid changes of the cinereous vulture **Table S15** Unique amino acid changes of the Accipitridae. **Table S16** Unique amino acid changes on sites between Accipitridae and Cathartidae of the digestive system-related proteins. **Table S17** Unique amino acid changes on sites between Accipitridae and Cathartidae of the gastric acid secretion-related proteins. **Table S18** Statistics regarding whole-transcriptome sequences and unigene construction. **Table S19** GO analysis for the blood transcriptome of the cinereous vulture. **Table S20** KEGG pathway analysis for the blood transcriptome of the cinereous vulture. **Table S21** Gene expression in the cinereous vulture compared to the other avian species. **Table S22** Immune related genes expression in the cinereous vulture compared to the other avian species. (XLSX 635 kb) Additional file 2: Fig. S1. Estimation of genome size using 17-mers. **Fig. S2** The divergence time of avian species. **Fig. S3** Specific amino acid changes in the gastric acid secretion associated genes. **Fig. S4** Species distribution of BLASTx top hits of cinereous vulture transcripts. **Fig. S5** NR protein database properties for the assembled unigenes. **Fig. S6** Gene Ontology classifications of cinereous vulture unigenes. **Fig. S7** Amino-acid sequence comparison of toll-like receptor 1 of the Griffon and cinereous vultures. (DOCX 2228 kb)
