# Supplementary material for: The first whole genome and transcriptome of the cinereous vulture reveals adaptation in the gastric and immune defense systems and possible convergent evolution between the Old and New World vultures
Source: Genome Biol. 2015 Oct 21;16:215. doi: 10.1186/s13059-015-0780-4 (PMC4618389; doi:10.1186/s13059-015-0780-4)
Supplement: Additional file 2: Fig. S1. — Estimation of genome size using 17-mers. Fig. S2 The divergence time of avian species. Fig. S3 Specific amino acid changes in the gastric acid secretion associated genes. Fig. S4 Species distribution of BLASTx top hits of cinereous vulture transcripts. Fig. S5 NR protein database properties for the assembled unigenes. Fig. S6 Gene Ontology classifications of cinereous vulture unigenes. Fig. S7 Amino-acid sequence comparison of toll-like receptor 1 of the Griffon and cinereous vultures. (DOCX 2228 kb) [file 13059_2015_780_MOESM2_ESM.docx]

**Fig. S1 Estimation of genome size using 17-mers.**


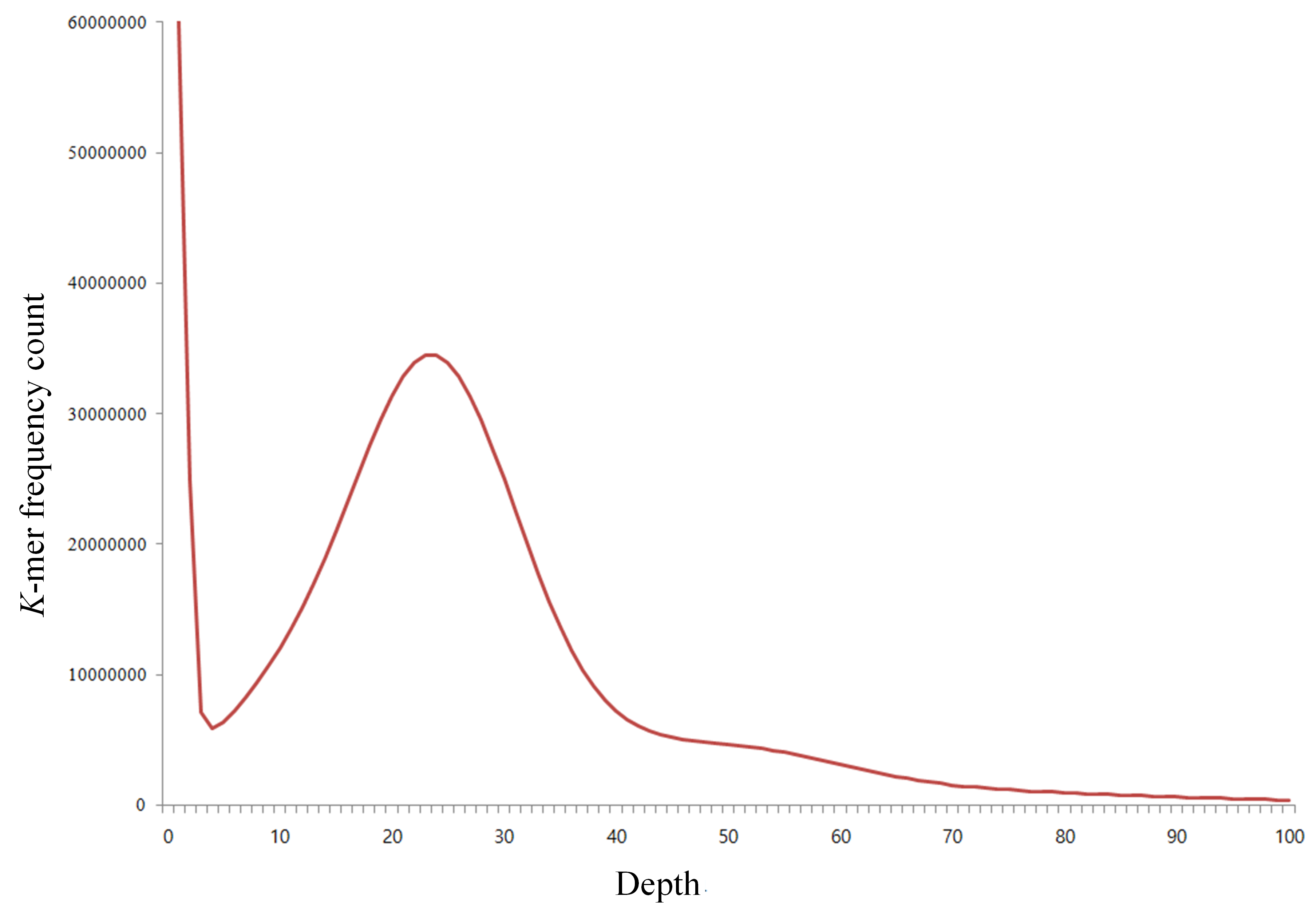


The x-axis represents depth, and the y-axis represents proportion, as calculated by the frequency at a given depth divided by the total frequency at all depths.


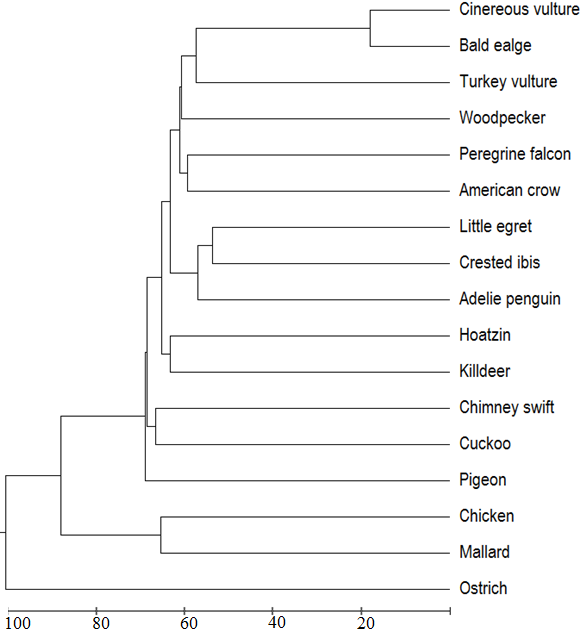


Fig. S2 The divergence time of avian species. The divergence time of the cinereous vulture and other avian based on four-fold degenerate sites.


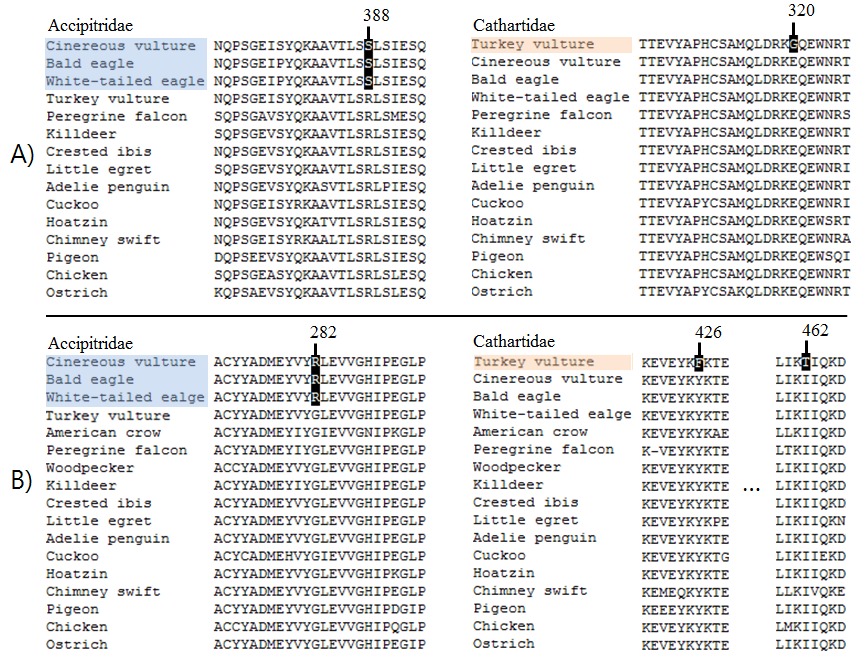
 Fig. S3. Specific amino acid changes in the gastric acid secretion associated genes. A Accipitridae- or Cathartidae-specific amino acid changes in the KCNJ16 protein. The specific amino acid changes are shown in the black rectangle. B The specific amino acid changes in the SLC26A7 protein.


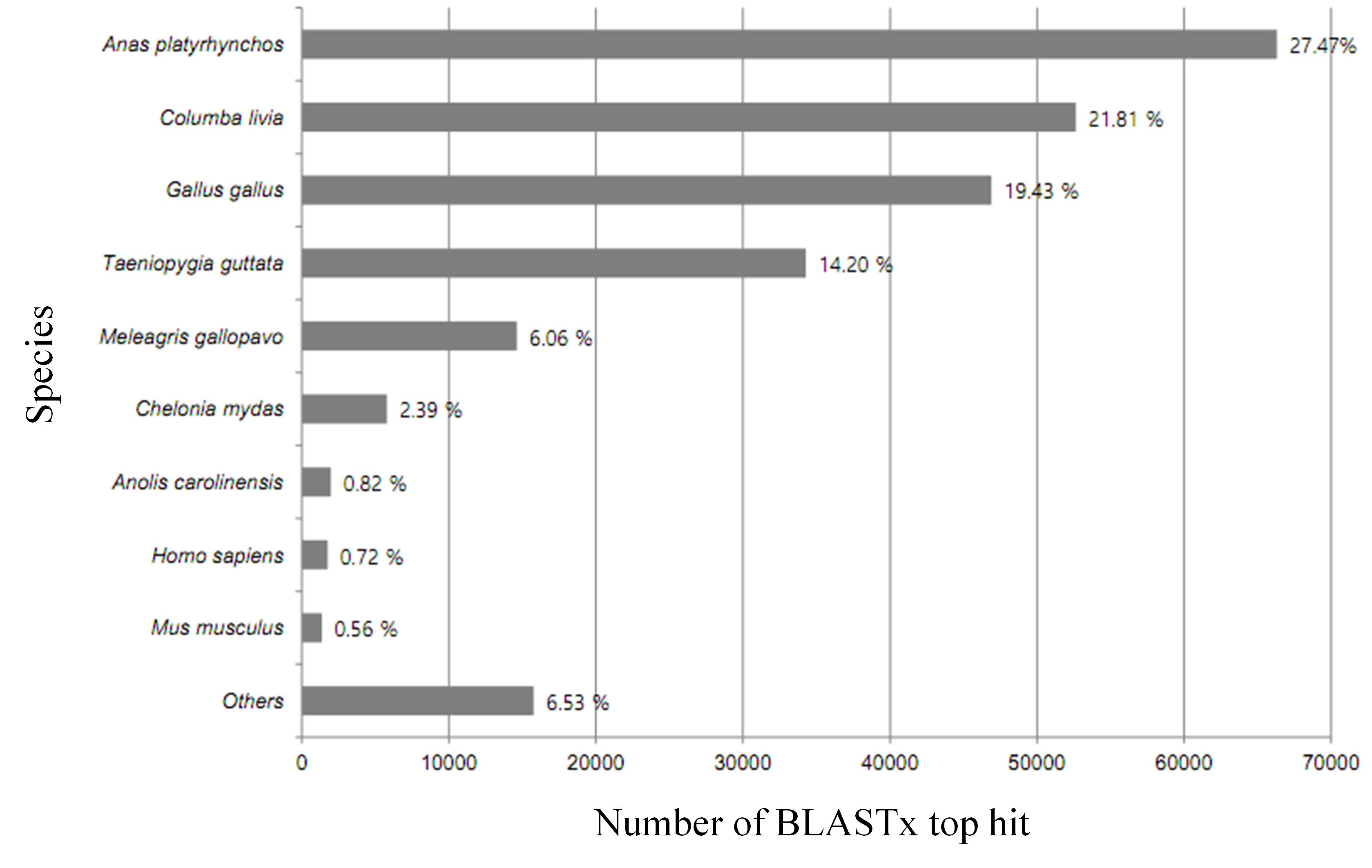


# **Fig. S4 Species distribution of BLASTx top hits of cinereous vulture transcripts.** Transcripts of the cinereous vulture exhibited the highest sequence similarity to those of *Anas platyrhynchos*. The percentages indicate the proportions of matched unigenes out of the total unigenes for each species.


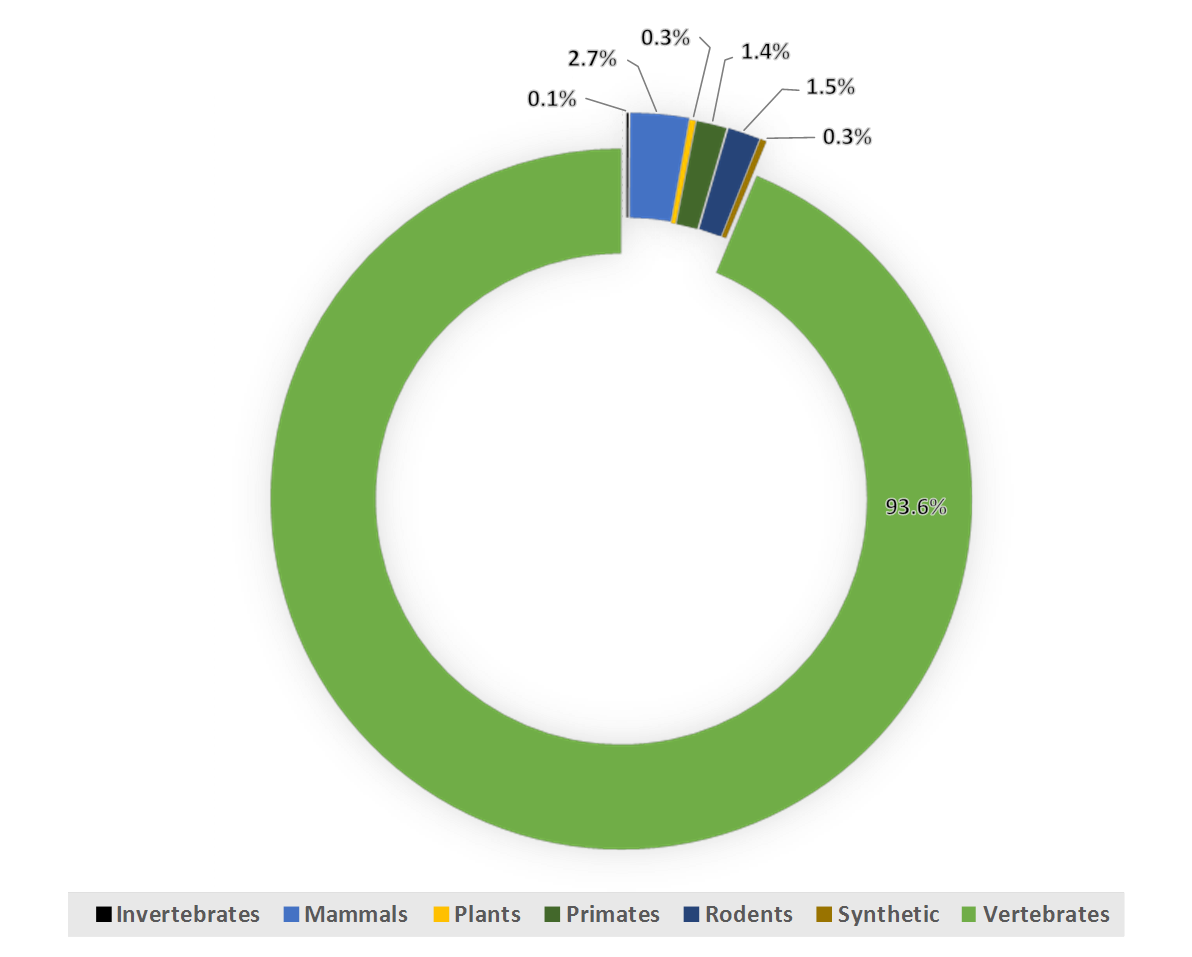


## **Fig. S5 NR protein database properties for the assembled unigenes.** Species distribution of the top BLASTx hits for the assembled unigenes, with a cutoff of 1E-5. Groups of species with very low percentages (bacteria: 0.01 %, viruses: 0.05 %, and environmental samples: 0.002 %) were omitted from this figure.


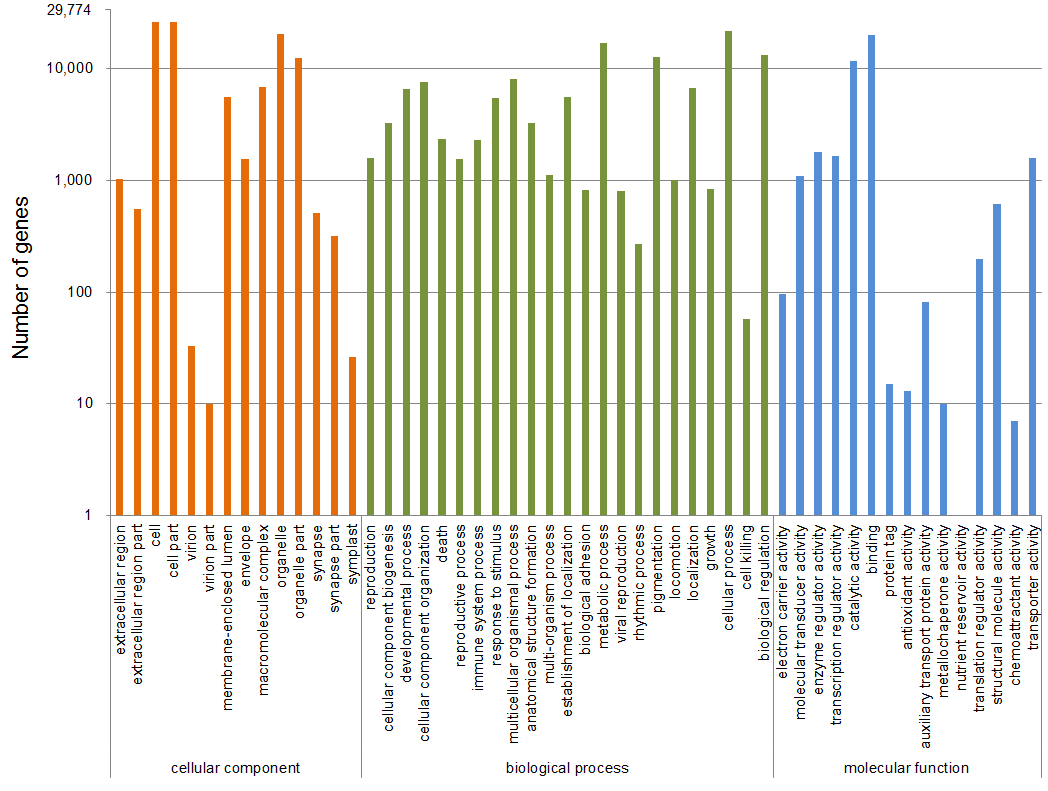


Fig. S6 Gene Ontology classifications of cinereous vulture unigenes. In total, 29,774 unigenes were assigned Gene Ontology terms in 52 functional groups in three categories (cellular component, biological process, and molecular function).


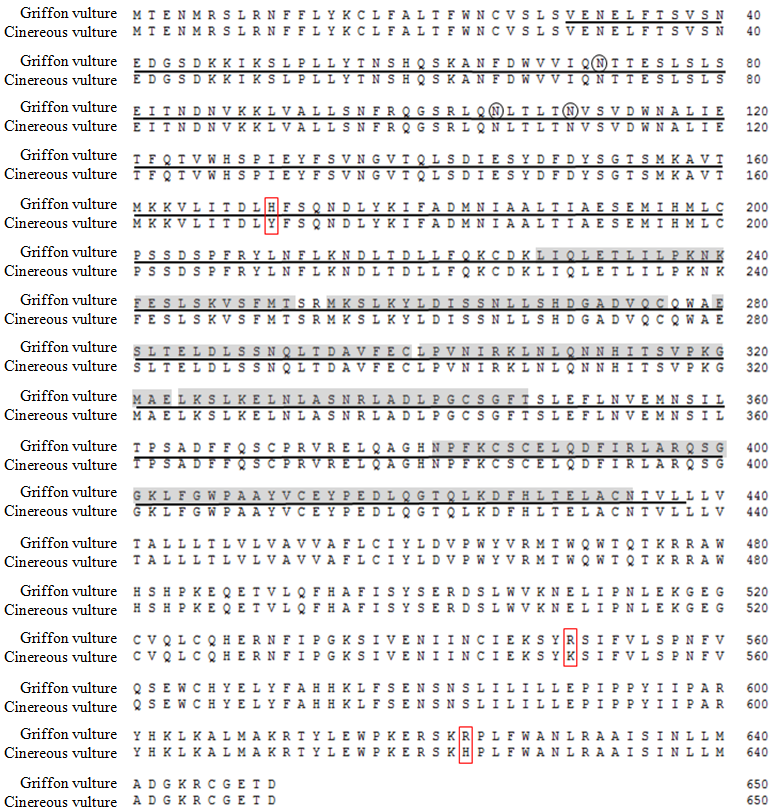


## **Fig. S7 Amino-acid sequence comparison of toll-like receptor 1 of the griffon and cinereous vultures.** Leucine-rich repeats motifs are shaded. Potential *N*-glycosylation sites are circled. Ectodomain is underlined. Red boxes identify sites where the two vulture sequences differ.
